# Yoda1 and phosphatidylserine exposure in red cells from patients with sickle cell anaemia

**DOI:** 10.1038/s41598-020-76979-2

**Published:** 2020-11-18

**Authors:** R. Wadud, A. Hannemann, D. C. Rees, J. N. Brewin, J. S. Gibson

**Affiliations:** 1 Department of Veterinary Medicine, Madingley Road, Cambridge, CB3 0ES UK; 2 Department of Paediatric Haematology, King’s College Hospital, Denmark Hill, London, SE5 5RL UK

**Keywords:** Physiology, Cardiovascular biology, Cardiovascular diseases, Peripheral vascular disease

## Abstract

Phosphatidylserine (PS) exposure is increased in red cells from sickle cell anaemia (SCA) patients. Externalised PS is prothrombotic and attractive to phagocytes and activated endothelial cells and thus contributes to the anaemic and ischaemic complications of SCA. The mechanism of PS exposure remains uncertain but it can follow increased intracellular Ca^2+^ concentration ([Ca^2+^]_i_). Normally, [Ca^2+^]_i_ is maintained at very low levels but in sickle cells, Ca^2+^ permeability is increased, especially following deoxygenation and sickling, mediated by a pathway sometimes called P_sickle_. The molecular identity of P_sickle_ is also unclear but recent work has implicated the mechanosensitive channel, PIEZO1. We used Yoda1, an PIEZO1 agonist, to investigate its role in sickle cells. Yoda1 caused an increase in [Ca^2+^]_i_ and PS exposure, which was inhibited by its antagonist Dooku1 and the PIEZO1 inhibitor GsMTx4, consistent with functional PIEZO1. However, PS exposure did not necessitate an increase in [Ca^2+^]_i_. Two PKC inhibitors were also tested, chelerytherine chloride and calphostin C. Both reduced PS exposure whilst chelerytherine chloride also reduced Yoda1-induced increases in [Ca^2+^]_i_. Findings are therefore consistent with the presence of PIEZO1 in sickle cells, able to mediate Ca^2+^ entry but that PKC was also involved in both Ca^2+^ entry and PS exposure.

## Introduction

As in other cells, the aminophospholipid phosphatidylserine (PS) of red cells is usually located on the inner leaflet of the membrane bilayer. This is important because externalised PS is prothrombotic, and also attractive to macrophages and activated endothelial cells^1^. PS exposure may thus be associated with red cell death and blockage of the microvasculature. Normally less than 1% red cells are positive for externalised PS. Notwithstanding, increased PS exposure does occur in healthy red cells during their normal ageing process^2–4^. In addition, however, red cells from patients with sickle cell anaemia (SCA) show less PS asymmetry than those from normal individuals, such that a high, but variable, proportion (c.1–10%), are positive for exposed PS^5,6^. Loss of the asymmetric distribution of this aminophospholipid may thereby contribute to both the chronic anaemia and acute ischaemic episodes characteristic of the morbidity of SCA.

Movement of PS across the red cell membrane is dependent mainly on three transport systems: (i) an ATP-dependent aminophospholipid translocase (or flippase) which moves PS from outer to inner leaflet using energy provided by ATP hydrolysis; (ii) a Ca^2+^-activated scramblase which rapidly moves PS in both directions; and (iii) a floppase which moves PS from inner to outer leaflet^7^. PS exposure requires both inhibition of the flippase together with activation of the scramblase^1,8^. Both of these may be precipitated by increases in intracellular Ca^2+^ concentration ([Ca^2+^]_i_) which under normal circumstances is kept at very low levels, around 30 nM, in comparison with a free plasma Ca^2+^ at a concentration some five orders of magnitude higher (at about 1.1–1.2 mM). Maintenance of these low levels of [Ca^2+^]_i_ is dependent upon the activity of a high capacity plasma membrane Ca^2+^ pump or ATPase (PMCA) coupled with a very low resting permeability to Ca^2+^^9^. As well as promoting PS exposure, elevation of Ca^2+^ is central to a number of other deleterious consequences which compromise red cell survival—including activation of the Ca^2+^-activated K^+^ channel (or Gárdos channel^10^) and of Ca^2+^-dependent proteases.

Red cells from SCA patients contain a mutated form of Hb, HbS, instead of the normal adult HbA. In HbS, a single base substitution results in the exchange of glutamic acid by valine at the sixth amino acid residue of the β chain^11^. Replacement of this negatively charged amino acid with a neutral one enables neighbouring molecules of deoxygenated HbS to adhere, forming long, rigid polymers which distort red cell shape, causing the sickling shape change. HbS-containing red cells also have a markedly abnormal cation permeability^12,13^. This is especially true for K^+^. There are three transport systems, which are either absent from or kept at low activity in red cells from normal individuals, but which are upregulated or abnormally active in sickle cells: the KCl cotransporter (KCC), a deoxygenation-induced cation conductance (sometimes called P_sickle_) and the Ca^2+^-activated K^+^ channel or Gárdos channel^13^. These three systems mediate net ion loss, with water following osmotically, thus raising the concentration of intracellular HbS, the abnormal Hb found in sickle cells. These events are relevant to pathogenesis as the lag time to polymerisation of HbS upon deoxygenation is highly dependent on its concentration. A lag inversely proportional to [HbS]^15-30^ is often quoted^14^. Small solute loss through these transporters may therefore markedly encourage sickling and a catalogue of damaging sequelae, contributing to the pathogenesis of SCA.

One of these cation permeablities, P_sickle_, is particularly significant for PS externalisation. The P_sickle_ pathway is activated by HbS deoxygenation, polymerisation and the sickling shape change^15^, and as well as making the cell leaky to univalent cations, it also mediates entry of Ca^2+^^16^. If [Ca^2+^]_i_ approaches a few hundred nanomolar, the co-ordinated inhibition of the flippase and activation of the scramblase is initiated. It is not surprising therefore that deoxygenation of sickle cells has been associated with increase in PS exposure^17^, which are both dependent on the presence of extracellular Ca^2+^ and which may be inhibited by intracellular Ca^2+^ chelation^3,18^.

With its proposed central role in SCA pathogenesis, it is understandable that considerable attention has been focused on regulation of P_sickle_ and identification of potential therapeutic inhibitors^16,19^. Notwithstanding its significance, however, the molecular identity of P_sickle_ still remains uncertain. Various pathways for mediation of P_sickle_ activity have been proposed, ranging from a simple disintegrity of the plasma membrane, to specific protein-mediated pathways including VDAC, NMDA receptors, TRPV channels as well as others^20^. Latterly attention has focused on PIEZO1^21^. PIEZO1 is a very large, multimeric channel with mechanosensitive properties. It is found in many tissues including red cells in which it has been proposed as a Ca^2+^ channel. There are two compelling pieces of evidence. Thus several ways of distorting the shape of normal red cells, for example shear stress, activate a Ca^2+^ entry process. In addition, gain-of-function mutations in PIEZO1 have been found in patients with hereditary xerocytosis^22–24^ and stomatocytosis^25–27^ with a proposed mechanism of increased Ca^2+^ entry causing aberrant Gárdos channel activation and therefore K^+^ loss and dehydration. It is also notable that a common PIEZO1 mutation is present in the African population^28^ although this mutation did not appear to correlate with P_sickle_ activity^29^. P_sickle_ has long thought to be unique to red cells from SCA patients, which is odd given that the underlying mutation resides in the β globin gene, and not in any a membrane transport protein. Findings with PIEZO1 suggest that a P_sickle_ entity may therefore be present and functional in red cells of all people.

Recently, several pharmacological agonists/antagonists of PIEZO1 have been described, including Yoda1, Dooku1 and Jedi1^30–33^. These reagents are somewhat problematical in that they may not be specific for PIEZO1 and, at least one of them, Yoda1, has been found have effects via other mechanisms, notably changes in protein phosphorylation^34^. Notwithstanding, an obvious approach to investigate the involvement of PIEZO1 as a candidate for P_sickle_ was to test them on Ca^2+^ entry and PS exposure in red cells from SCA patients.

There have also been several reports implicating an important role for protein kinase C (PKC) in red cell PS exposure, possibly via opening of Ca^2+^ channels, but also possibly through a Ca^2+^-independent action^5,35,36^. Thus various PKC inhibitors reduce PS exposure, and some, but not all, also reduce Ca^2+^ entry in normal red cells.

In this report, we investigated the roles of Ca^2+^, PIEZO1 and PKC in PS exposure in sickle cells using agonists and inhibitors of PIEZO1, Ca^2+^ ionophores, and PKC antagonists. Results show that the PIEZO1 agonist Yoda1 stimulated Ca^2+^ entry and caused PS exposure in almost all red cells from SCA patients. An increase in [Ca^2+^]_i_, however, was not a pre-requisite for PS exposure. Rather Yoda1 appeared to act mainly via PKC in a Ca^2+^-dependent and Ca^2+^-independent mechanism. These results with PIEZO1 modulators confirm previous reports of the complexity of PS exposure in red cells including sickle cells and emphasise an important caveat that pharmacological reagents are often promiscuous in their effect.

## Methods

### Chemicals

Fluorescein isothiocyanate-conjugated lactadherin (LA-FITC) and phalloidin (FITC-phalloidin) came from Haematologic Technologies Inc. (Essex Junction, VT, USA), supplied by Cambridge Bioscience (Cambridge, UK). Anti-glycophorin A-PE from Becton Dickinson Biosciences (CA, USA) came from Enzyme Research Laboratories (Swansea, UK). Bromo-A23187, 4-(2-hydroxyethyl)-1-piperazineethanesulfonic acid (HEPES) and Fluo-4-AM came from Calbiochem (Merck, Darmstadt, Germany). Yoda1, Dooku1 and GsMTx4 were supplied by Tocris Bioscience (Abingdon, UK), Glixx Laboratories Inc (Hopkinton, MA, USA) and AbCam (Cambridge, UK); and AbCam also supplied the PKC inhibitors chelerytherine chloride and calphostin C. All other chemicals were supplied by Sigma-Aldrich Co. (Poole, Dorset, UK).

### Sample collection and handling

Blood samples were taken with informed consent from patients homozygous for sickle cell anaemia (SCA), HbSS genotype, using the anticoagulant EDTA. The study was approved by the National Health Service (NHS) National Research Ethics Committee (NREC reference 16/LO/1309). For some experiments, once routine haematological assays had been completed, discarded and anonymised blood was used. All research was conducted with ethical approval and in accordance with the Helsinki Declaration of 1975, as revised in 2008.

### Solutions and red cell preparation

The standard salines, buffered with 4-(2-hydroxyethyl)piperazine-1-ethanesulfonic acid **(**HEPES), were either low K^+^ (LK-HBS) or high K^+^ (HK-HBS). For LK-HBS, saline comprised (in mM): NaCl 145, KCl 5, MgCl_2_ 0.15, inosine 10 and HEPES 10, (pH 7.4 at 37 °C; 290 ± 5 mOsm kg^−1^). For HK-HBS, salines were similar but with NaCl 54 mM and KCl 90 mM. For Ca^2+^ titration curves, additions of EGTA (2 mM) and concentrations of total [Ca^2+^] of 0, 1.35, 1.63, 1.72, 1.81, 1.85, 1.91 and 2.00 mM were used to clamp free extracellular [Ca^2+^] ([Ca^2+^]_o_) at 0, 0.1, 0.2, 0.3, 0.45, 0.6, 1 and 10 µM, respectively. Where required, red cells were permeabilised to Ca^2+^ with the ionophore bromo-A23187 (6 µM final). For oxidant challenge, a stock solution of *tert*-butyl hydroperoxide (*t*BHP) was made in LK-HBS. Exposed PS was labelled with LA-FITC (16 nM) in HK-HBS (pH 7.4 at room temperature, RT) containing 1 mM vanadate (LA-FITC binding buffer). To prepare red cells, whole blood was washed four times in HK-HBS (pH 7.4 at RT) to remove plasma and buffy coat. For experiments in which intracellular [Ca^2+^] ([Ca^2+^]_i_) was manipulated, the final two washes contained, in addition, EGTA (1 mM) to remove any contaminant Ca^2+^. Red cells were stored on ice until required. Haematocrit (Hct) was measured using Drabkin's reagent.

### Measurement of externalised PS using LA-FITC

To promote lipid scrambling, red cells were incubated in HK-HBS (0.5% Hct; 30 min, 37 °C) containing 2 mM EGTA at various [Ca^2+^]_o_s, using bromo-A23187 (6 µM) to permeabilise red cells to Ca^2+^, in the absence or presence of different oxidants or thiol modifiers. The activity of bromo-A23187 was abrogated by adding 0.4 mM Co^2+^. Red cells were then pelleted and resuspended in HK-HBS containing 1 mM vanadate (pH 7.4 at RT), diluted in LA-FITC binding buffer at 0.01% Hct, and incubated for 15 min in the dark at RT. Red cells were next pelleted (10 s at 16,100 g), washed once in HK-HBS, resuspended and kept on ice in the dark until flow cytometry analysis. LA-FITC was detected in the FL1 channel of a BD Accuri C6 flow cytometer using logarithmic gain (as for CM-H_2_-DCF fluorescence). The positive fluorescent gate was set using red cells unlabelled with LA-FITC. For each measurement 10,000 events were gated. PS positive cells were defined as all events falling within the preset FSC, SSC and positive fluorescent gates. See^18^ for detailed Methodology.

### Measurement of red cell membrane integrity

Red cells were treated with *t*BHP (0–1 mM) or Yoda1 in HK-HBS (0.5% Hct, pH 7.4 for 20 min at 37 °C), washed and resuspended in HK-HBS. The fluorophore phalloidin-iFluor 647 was added and washed cells were analysed by flow cytometry. Phalloidin labels intracellular actin found in the red cell cytoskeleton. The presence of positive cells indicates that the plasma membrane has been disrupted to allow entry of phalloidin to the cell interior. A positive signal therefore implies of loss of membrane integrity (cf^37^).

### Statistics

Results are presented as means ± SEM for blood samples of *n* different individuals. All experiments were carried out on paired samples so that control cells, and those treated with one or more inhibitor, were always carried out at the same time, using cells from the same blood donors. Where appropriate, comparisons were therefore made using 2-tailed Student's *t*-tests and p < 0.05 was considered as significant.

## Results

### Yoda1 and intracellular Ca^2+^

**Figure 1:**
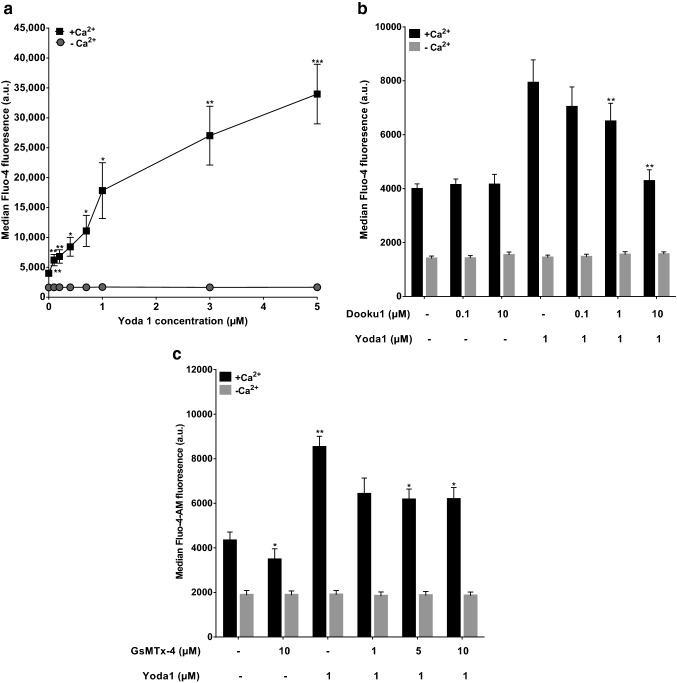
Effect of Yoda1 on intracellular Ca^2+^ concentration in red cells from patients with sickle cell anaemia (SCA). Red cells (0.5% Hct), pre-loaded with Fluo-4-AM (5 µM), were equilibrated in air and treated with Yoda1 (0.1 to 5 µM) for 20 min at 37 °C in the absence (EGTA 1 mM and 0 Ca^2+^) and presence of extracellular Ca^2+^ (1.1 mM). Intracellular Ca^2+^ was measured using Fluo-4. (**a**) Dose response of Yoda1 in the presence and absence of extracellular Ca^2+^. **p < 0.01, *p < 0.05 and ***p < 0.001, comparing Red cells treated with Yoda1 (at 0, 0.1, 0.2 and 3 µM; 0.4, 0.7 and 1 µM; and 5 µM, respectively) in the absence and presence of Ca^2+^. Symbols represent means ± SEM, n = 4. (**b**) Effect of Dooku1 on Yoda1-induced rise in intracellular Ca^2+^ concentration. Red cells were pre-incubated with Dooku1 (0.1 µM, 1 µM and 10 µM) for 10 min at 37 °C, following which they were treated with Yoda 1 (1 µM) for a further 20 min at 37 °C. **p < 0.01 comparing red cells in absence of Dooku1 and its presence. Symbols represent means ± SEM, n = 5. (**c**) Effect of GsMTx4 on Yoda1-induced rise in intracellular Ca^2+^ concentration. Red cells were pre-incubated with GSMTx4 (1 µM, 5 µM and 10 µM) for 10 min at 37 °C following which they were treated with Yoda 1 (1 µM) for a further 20 min at 37 °C. *p < 0.05 and **p < 0.01, respectively, comparing either red cells in the presence of Yoda1 (1 µM) alone with those pre-incubated GsMTx4 (5 µM and 10 µM) in the presence of extracellular Ca^2+^, or those treated with DMSO only with those exposed in addition to Yoda (1 µM). Histograms represent means ± SEM, n = 5.

In the presence of extracellular Ca^2+^, Yoda1 caused a concentration-dependent elevation in [Ca^2+^]_i_ (Fig. 1a). This effect became significant at a concentration of 100 nM (*p* < 0.01) whilst [Ca^2+^]_i_ continued to increase up to the maximum concentration used (5 µM). In the absence of extracellular Ca^2+^, as anticipated, Yoda1 had no effect on [Ca^2+^]_i_ (Fig. 1). Dooku1, an antagonistic analogue of Yoda1 which probably interferes with the binding of Yoda1 to its target^31^, inhibited the Yoda1-induced rise in [Ca^2+^]_i_ abolishing the change at a concentration of 10 µM (Fig. 1b). The spider venom GsMTx4 (up to 10 µM), which probably inhibits PIEZO1 through alterations in stress of the lipid bilayer, similarly reduced the stimulatory effect of Yoda1 on elevation of [Ca^2+^]_i_, although its effect was smaller (Fig. 1c). GsMTx4 also slightly but significantly reduced the resting [Ca^2+^]_i_ in the absence of Yoda1 which would indicate a constitutively active pathway sensitive to this inhibitor. By contrast, a second analogue of Yoda1, Jedi, was without effect (data not shown). These findings are consistent with a Yoda1-induced stimulation of Ca^2+^ entry across the plasma membrane. As Yoda1, Dooku1 and GsMTx4 are all considered to interact with PIEZO1, these results are compatible with PIEZO1-mediated Ca^2+^ entry and that the majority of red cells can react to mediate Ca^2+^ increase.

### Yoda1 and phosphatidylserine exposure

**Figure 2:**
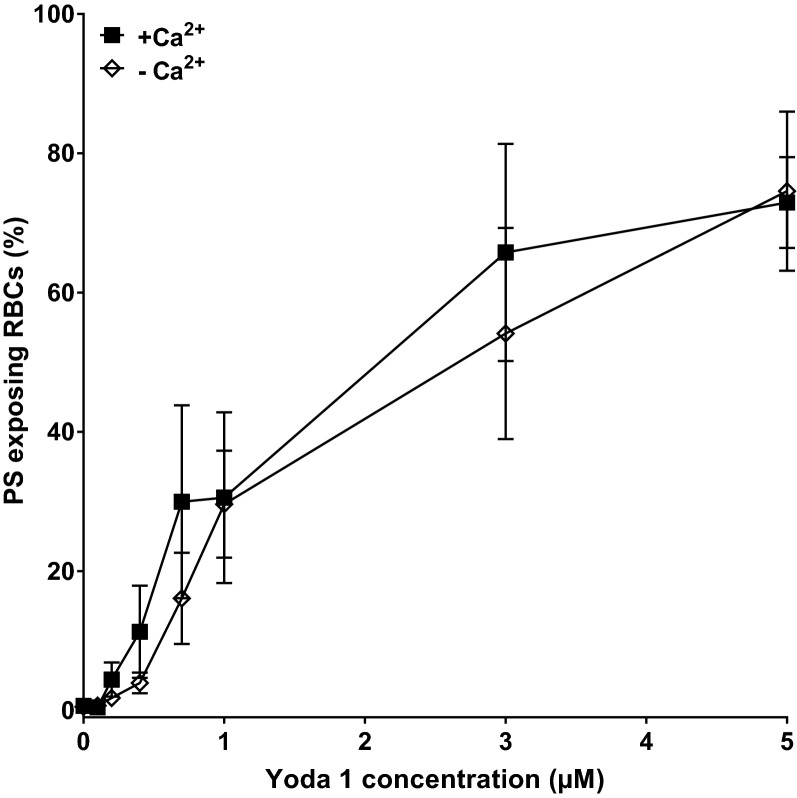
Effect of Yoda1 on phosphatidylserine (PS) exposure in red cells from SCA patients. Red cells (0.5% Hct) were equilibrated in air with Yoda1 for 20 min at 37 °C in the absence (EGTA 1 mM and 0 Ca^2+^) and presence (1.1 mM) of extracelluar Ca^2+^. They were then labelled with LA-FITC to detect exposed PS using flow cytometry. There was no significant difference in PS exposure without or with extracellular Ca^2+^. Symbols represent means ± SEM, n = 4.

In the next series of experiments, the effect of Yoda1 on phosphatidylserine (PS) exposure in red cells from SCA patients was investigated, first in the absence of Ca^2+^ ionophore. As expected from its effects on [Ca^2+^]_I_, Yoda1 treatment resulted in increased PS exposure in the majority (about 80%) of the red cell population (Fig. 2) at similar concentrations to those required to mediate elevation in intracellular Ca^2+^, with a significant increase in PS exposure at a Yoda1 concentration of 100 nM (*p* < 0.05).

Whilst these findings suggested that Ca^2+^ entry was directly responsible for the lipid scrambling, there was, however, little, or no, inhibitory effect on Yoda1-induced PS exposure when red cells were incubated in Ca^2+^-free saline (Fig. 2), in which circumstances [Ca^2+^]_i_ remained at control levels (cf Fig. 1). At concentrations of Yoda1 ≥ 0.5 µM, PS exposure was identical in the presence or absence of extracellular Ca^2+^. At lower concentrations, there was a suggestion, albeit non-significant, that the presence of extracellular Ca^2+^ resulted in higher levels of PS exposure (Fig. 2).

**Figure 3:**
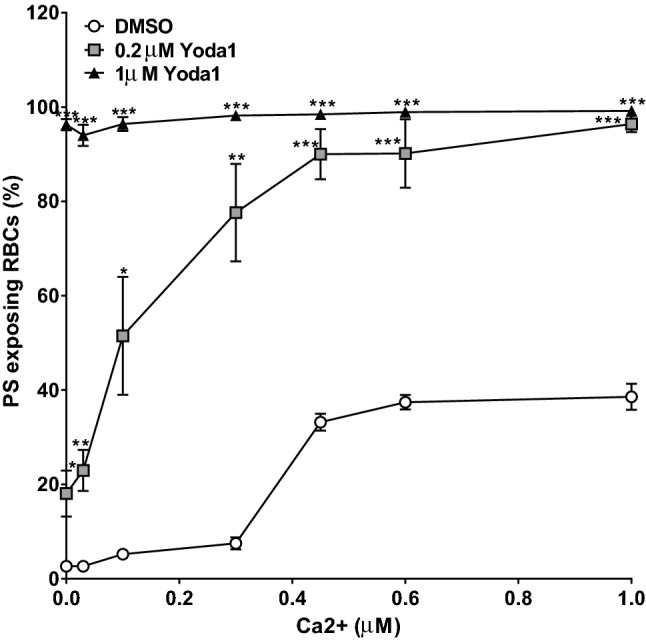
Effect of Yoda1 and Ca^2+^ on PS exposure in red cells from SCA patients. Red cells (0.5% Hct) were equilibrated in air with Yoda1 (0.2 µM and 1 µM) at different extracellular Ca^2+^ concentrations (0, 0.03 µM, 0.1 µM, 0.3 µM, 0.45 µM, 0.6 µM, 1 µM) for 30 min at 37 °C, following which red cell aliquots were removed and exposed PS labelled with LA-FITC. *p < 0.05, **p < 0.01 and ***p < 0.001 comparing red cells in the absence and presence of Yoda1 (0.2 µM) at free extracellular Ca^2+^s of 0 µM and 0.1 µM; 0.03 µM and 0.3 µM; and 0.45 µM, 0.6 µM and 1 µM, respectively; 2 mM EGTA). EC_50_s for extracellular Ca^2+^ were 0.37 µM, 0.13 µM and 0.18 µM for DMSO, 0.2 µM and 1 µM Yoda1, respectively. Symbols represent means ± SEM, n = 5.

The effect of Ca^2+^ was investigated in more detail in red cells treated with the ionophore bromo-A23187 (6 µM) to clamp [Ca^2+^]_i_ over a range of concentrations from 0 to 1.0 µM. Results compared red cells exposed to Yoda1 at two concentrations, 0.2 and 1.0 µM with those incubated in its absence (Fig. 3). Without Yoda1, PS exposure increased as [Ca^2+^]_i_ was elevated with an EC_50_ of 0.37 µM and with a plateau reached at about 0.45 µM at which about 40% cells were positive for externalised PS, consistent with findings from previous work^3,15,34^. At the lower concentration of Yoda1 (0.2 µM), PS exposure was also Ca^2+^ dependent but the concentration dependence was altered to significantly lower values (EC_50_ 0.18). Even in the absence of Ca^2+^, with 0.2 µM Yoda1 PS exposure reached about 20% (*p* < 0.05 cf cells without Yoda1). In addition, almost all cells were positive for external PS with Ca^2+^ of ≥ 0.5 µM (cf about 35% in the absence of Yoda1 at the highest [Ca^2+^] tested). With the higher concentration of Yoda1 (1.0 µM), PS exposure became Ca^2+^-independent and all cells were positive for PS over the whole Ca^2+^ titration, including cells incubated in the absence of Ca^2+^.

**Figure 4:**
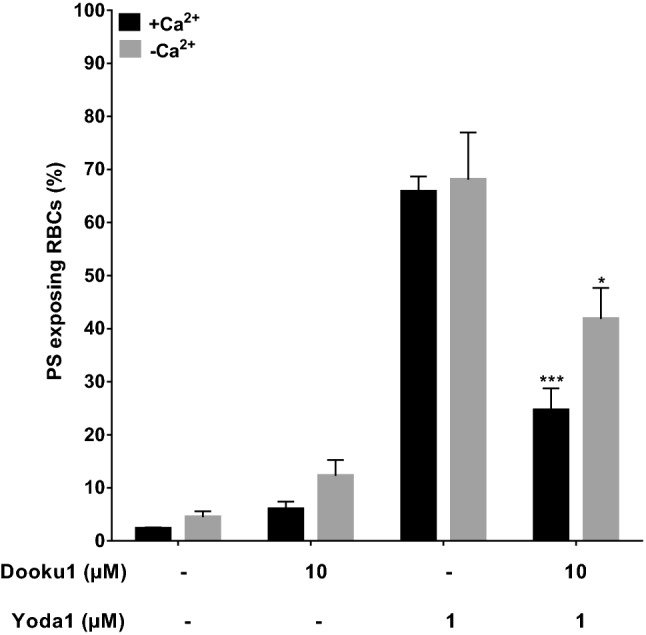
Effect of Dooku1 on Yoda1-induced PS exposure in red cells from SCA patients. Red cells (0.5% Hct) were equilibrated in air in the absence (EGTA 1 mM and 0 Ca^2+^) or presence (1.1 mM) of Ca^2+^. Red cells were initially incubated for 10 min with Dooku1 (10 µM) and then treated with Yoda1 (1 µM) for a further 30 min, all at 37 °C. Red cell aliquots were then removed and exposed PS labelled with LA-FITC. *p < 0.05 and ***p < 0.001 comparing red cells in the presence of Yoda1 alone and those pre-incubated Dooku1 (10 µM) in the absence of extracellular Ca^2+^ or in its presence, respectively. Histograms represent means ± SEM, n = 6.

The effects of Dooku1 and GsMTx4 on PS exposure were also tested in combination with Yoda1. Results were similar to the findings on intracellular Ca^2+^ levels. Thus Dooku1 (up to 10 µM) reduced Yoda1-induced PS exposure, in both the presence and absence of Ca^2+^ (Fig. 4), consistent with its action as a Yoda1 antagonist. In contrast, GsMTx4 (up to 5 µM) had variable effects on PS exposure (data not shown).

### Yoda1 and membrane integrity

**Figure 5:**
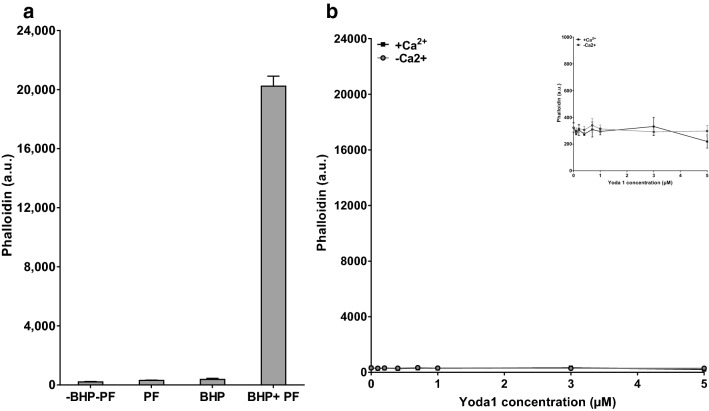
Phalloidin and membrane integrity of red cells from SCA patients. (**a**) Effect of the oxidant *tert* butyl hydroperoxide: Red cells were incubated for 20 min without (− *t*BHP) or with *tert* butyl hydroperoxide (+ *t*BHP, 0.78 mM) and then exposed to phalloidin-iFluor 647 (1:400; + PF) or left unlabelled (− PF). Phalloidin-iFluor 647 fluorescence is given in arbitrary units (a.u.). Histograms represent means ± SEM, n = 5. (**b**) Effect of Yoda1: Red cells (0.5% Hct) were incubated in air for for 20 min at 37 °C without or with Yoda1 (up to 5 μM) in the absence (1 mM EGTA and 0 mM Ca^2+^; − Ca^2+^) or presence (extracellular Ca^2+^ 1.1 mM; + Ca^2+^) of extracellular Ca^2+^. They were then exposed to phalloidin-iFluor 647 (1:400). Phalloidin-iFluor 647 fluorescence is given in arbitrary units (a.u.), detected using flow cytometry using the same scale as Fig. 7a (peak fluorescence 24,000) and also at higher scale (insert, peak fluorescence 1000). The percentage of red cells staining positive ofr phalloidin were 52 ± 14% in cells treated with *t*BHF and 2–3 ± 1% for all other conditions. Symbols represent means ± SEM, n = 4.

The above findings showed that Yoda1-induced PS labelling was Ca^2+^-dependent at lower concentrations but, unexpectedly became Ca^2+^-independent at higher concentrations. One possibility to account for these observations was a breakdown in the lipid bilayer caused by higher Yoda1 concentrations, such that the fluorescent PS label (FITC-lactadherin) had access to phospholipids at both the inner, as well as the outer, leaflet. Results consistent with this hypothesis have been previously obtained using the oxidant *tert* butyl hydroperoxide^37^. If FITC-lactadherin had been able to access the inside of the lipid bilayer, positively labelled cells would be present in the absence of PS externalisation. To ascertain whether this possibility had occurred, red cells were exposed to fluorescently-labelled phalloidin (phalloidin-iFluor 647) which binds to intracellular actin, but can only gain access to its target if the membrane integrity is disrupted. In a control experiment, as expected, phalloidin-iFluor 647 was unable to label untreated red cells (Fig. 5). Following exposure to the oxidant *tert* butyl hydroperoxide (*t*BHP) at concentrations which disrupt the lipid bilayer, red cells stained positively with phalloidin-iFluor 647 due to the membrane damage (Fig. 5a). Yoda1-treated red cells, however, were not labelled with phalloidin-iFluor 647 at concentrations up to 5 µM (Fig. 5b), suggesting that the red cell membrane remained intact.

### Yoda1 and protein kinase C

From the above, it would therefore appear that Yoda1 did indeed induce PS exposure, but that neither the presence of extracellular Ca^2+^, nor a rise in intracellular [Ca^2+^]_i_, were required. Activation of PIEZO1 by Yoda1 with ensuing Ca^2+^ influx across the red cell membrane was therefore not a prerequisite for lipid scrambling. Rather there must be some alternative mechanism of action of Yoda1. Previously, Yoda1 has been shown to activate protein kinases in other tissues^34^, and there is also strong evidence of a role for protein kinase C (PKC) in PS exposure^2,35,36^, either via Ca^2+^ entry through cation channels or via a Ca^2+^-independent action. The effect of Yoda1 in sickle cells was therefore examined in combination with inhibitors of PKC, chelerytherine chloride which inhibits the active phosphorylation site of PKC, and calphostin C, which is an irreversible inhibitor of the diacylglyceraldehye-binding site.

**Figure 6:**
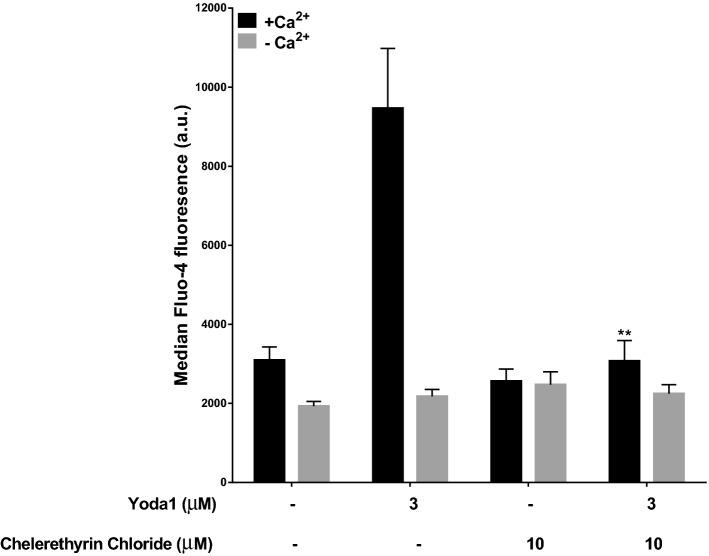
Effect of chelerytherine chloride on Yoda1-induced rise in intracellular Ca^2+^ concentration in red cells from SCA patients. Red cells (0.5% Hct) were equilibrated in air and pre-incubated with chelerethyrin chloride (10 µM) for 10 min following which they were treated with Yoda1 (1 µM and 3 µM) for a further 20 min, all at 37 °C, in the absence (EGTA 1 mM and 0 Ca^2+^) or presence (1.1 mM) of extracellular Ca^2+^. Red cell aliquots were then removed and exposed PS labelled with LA-FITC. **p < 0.01 comparing red cells in presence of Yoda1 (1 µM) alone and in combination with pre-incubated chelerythrine chloride (10 µM) in the absence (− Ca^2+^) and presence (+ Ca^2+^) of Ca^2+^. Histograms represent means ± SEM, n = 6.

The effect of chelerethyrine chloride on Yoda1-induced increase in [Ca^2+^]_i_ is shown in Fig. 6. As before, in the absence of extracellular Ca^2+^, Yoda1 (3 μM) had no effect on intracellular Ca^2+^ levels whilst in the presence of extracellular Ca^2+^, an increase in [Ca^2+^]_I_ was observed. This rise in Ca^2+^ was abolished by chelerethyrine chloride (10 µM), such that values were unchanged compared to cells incubated in the absence of Yoda1.

**Figure 7:**
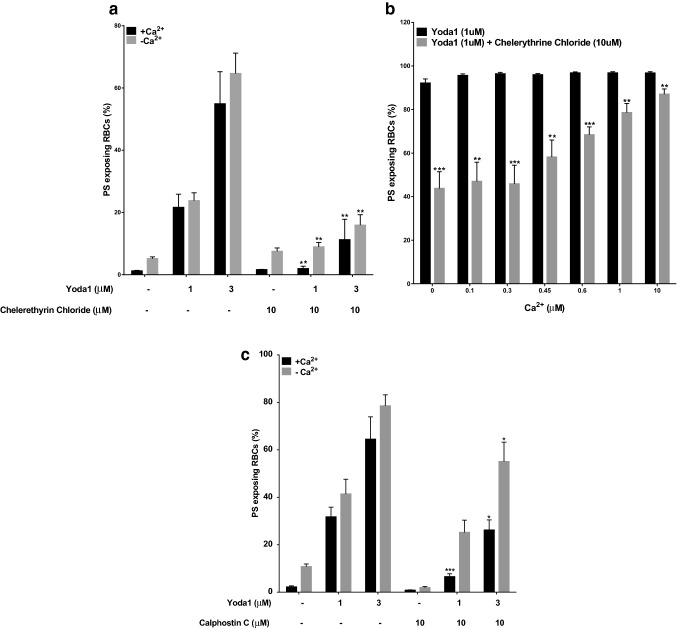
Effect of inhibitors of protein kinase C on PS exposure in red cells from SCA patients. (**a**) Effect of chelerytherine chloride on Yoda1-induced PS exposure: Red cells (0.5% Hct), pre-loaded with Fluo-4AM (5 µM), were equilibrated in air and pre-incubated with chelerethyrin chloride (10 µM) for 10 min following which they were treated with Yoda1 (3 µM) for 20 min, all at 37 °C, in the absence (EGTA 1 mM and 0 Ca^2+^) or presence (1.1 mM) of extracellular Ca^2+^. **p < 0.01 comparing Red cells in the presence of Yoda1 alone (3 µM) and in combination with chelerythrine chloride (10 µM). Histograms represent means ± SEM, n = 6. (**b**) Effect of chelerytherine chloride and Yoda1 in Ca^2+^-clamped Red cells: Red cells (0.5% Hct) were equilibrated in air and treated with Yoda1 (1 µM) at different extracellular Ca^2+^s for 30 min at 37 °C. Red cells (0.5% Hct) were equilibrated in air and pre-incubated with chelerythrine chloride (10 µM) at different extracellular Ca^2+^s for 10 min, following which they were treated with Yoda1 (1 µM) for 20 min, all at 37 °C. Red cells aliquots were then removed and exposed PS labelled with LA-FITC. ***p < 0.001 and **p < 0.01 comparing red cells in the presence of Yoda1 alone (1 µM) and in combination with chelerythrine chloride (10 µM) at extracellular Ca^2+^s of 0 µM, 0.3 µM and 0.6 µM and 0.1 µM, 0.45 µM, 1 µM and 10 µM, respectively. Histograms represent means ± SEM, n = 6. (**c**) Effect of calphosin C on Yoda1-induced PS exposure: Red cells (0.5% Hct) were equilibrated in air and pre-incubated with calphostin C (10 µM) for 10 min following which they were treated with Yoda1 (1 µM and 3 µM) for 20 min, all at 37 °C, in the absence (EGTA 1 mM and 0 Ca^2+^) or presence (1.1 mM) of extracellular Ca^2+^. RBC aliquots were then removed and exposed PS labelled with LA-FITC. ***p < 0.001 and *p < 0.05 comparing Red cells in the presence of Yoda1 alone (1 µM) and in combination with calphostin C (10 µM) in the presence (1.1 mM) or absence (EGTA) of extracellular Ca^2+^, respectively. Histograms represent means ± SEM, n = 4.

When investigating the effect of chelerytherine chloride (10 µM) on PS exposure, on its own, this PKC inhibitor had no effect. When combined with Yoda1 (3 µM), however, the Yoda1-induced PS exposure was completely inhibited at 1 µM Yoda1 and substantially reduced at 3 µM Yoda1 (Fig. 7a). This figure also shows that the stimulatory effect of Yoda1 on PS exposure did not require Ca^2+^.

The effect of chelerytherine chloride on PS exposure on Ca^2+^-clamped red cells was then investigated (Fig. 7b). At low Ca^2+^ concentrations, chelerytherine chloride substantially inhibited the Yoda1-induced PS exposure, by about 50%, but inhibition was reduced as the concentration of Ca^2+^ was increased such that at a [Ca^2+^] of 10 µM inhibition was minimal although significant (about 10% reduction, p < 0.01). These findings suggest that at low Ca^2+^ levels Yoda1-induced PS exposure is PKC-dependent but that at higher values it becomes PKC-independent.

In the last series of experiments, the effect of a second PKC inhibitor, calphostin C, was investigated. The effect of calphostin C on Yoda1-induced increase in PS exposure was determined (Fig. 7c). At lower Yoda1 concentrations (1 µM), calphostin C (10 µM) had a large inhibitory effect (about 70%, *p* < 0.05). Inhibition was still observed at the higher Yoda1 concentration (3 µM) though much reduced (by about 30%). These findings suggest that Yoda1-induced rise in Ca^2+^ can be mediated by PKC but that another mechanism is also present, presumably via stimulation of PIEZO1, whilst Yoda1-induced PS exposure, at least at low concentrations of Ca^2+^, is predominantly via PKC although high Ca^2+^ causes a PKC-independent PS exposure.

## Discussion

The effects of Yoda1, a pharmacological activator of PIEZO1, were investigated on intracellular [Ca^2+^] ([Ca^2+^]_i_) and phosphatidylserine (PS) exposure in red cells from patients with sickle cell anaemia (SCA).

Yoda1 caused a rise in [Ca^2+^]_i_, dependent on the presence of extracellular Ca^2+^, consistent with its increased entry across the plasma membrane (Fig. 1a). Findings that the PIEZO1 inhibitors, Dooku1 and GsMTx4, inhibited the elevation in [Ca^2+^]_i_ were consistent with this mechanosensitive channel being the likely target of Yoda1 for the Ca^2+^ rise (Figs. 1b,c,3). The majority of red cells responded to Yoda1 implying that most sickle cells express PIEZO1 in their membranes, notwithstanding their stochastic increase in cation permeability in response to deoxygenation and the sickling shape change^38^.

PKC inhibitors, however, also reduced the rise in intracellular Ca^2+^, making it unlikely that PIEZO1 was the sole, or even the main, target of Yoda1 and indicating that its pharmacology is more complicated (Figs. 6 and 7, and text). In the case of PS exposure, Yoda1-induced externalisation occurred in the presence, but also in the complete absence, of extracellular Ca^2+^, indicating that Yoda1 was able to stimulate PS exposure independent of a rise in [Ca^2+^]_I_ (Figs. 2 and 7). Notwithstanding that Yoda1 appears to act additionally via PKC, it clearly can interact with PIEZO1, as shown by patch clamp experiments^30^. It is therefore still possible that it similarly stimulates PIEZO1 in red cells. Whether it does awaits further confirmation in electrophysiological experiments. In addition, it was also noticeable that the extent of PS exposure in response to Yoda1, whilst always present, differed between different red cell samples, indicative of the marked heterogeneous behaviour of sickle cells observed across SCA patients.

Our previous work with the oxidant *tert* butyl hydroperoxide (*t*BHF) produced a caveat that some reagents can damage the membrane and allow access of the PS label to inside^37^. The highest Yoda1 concentrations tested, however, did not allow access of fluorescently-labelled phalloidin (phalloidin-iFluor 647). The findings shown in Fig. 5b clearly indicate the lack of permeability to phalloidin-iFluor 647 in Yoda1-treated red cells—there is no fluorescent labelling–whilst phalloidin could gain access following exposure to the oxidant *t*BHF (Fig. 5b). These findings negated the explanation of disintegrity of the red cell membrane following Yoda1 incubation. The results for PS labelling were not therefore due to Yoda1-induced membrane damage allowing access of FITC-lactadherin to PS present in the inner leaflet of the RBC membrane bilayer.

With respect to the two inhibitors of protein kinase C (PKC) tested, chelerytherine chloride reduced the Yoda1-induced increase in [Ca^2+^]_i_, consistent with Yoda1 acting also via a PKC-activated cation channel (Fig. 6). Both chelerytherine chloride and calphostin C also reduced Yoda1-induced PS exposure (Fig. 7a,c), also indicating an action partially via PKC. Notwithstanding, inhibition of PS exposure by chelerytherine chloride was attenuated as [Ca^2+^]_I_ was increased using a Ca^2+^ionophore (Fig. 7b), indicating an additional effect of intracellular Ca^2+^ independent of PKC, although it is also possible that PKC inhibition used here was incompleteat the concentration of chelerytherine chloride.

Previous work using phorbol myristate acetate (PMA), lysophosphatidic acid (LPA) and the Ca^2+^ ionophore A23187 together with chelerytherine chloride and calphostin C has also produced evidence for PKC-mediated PS exposure in normal and sickle cells, through both Ca^2+^-dependent and Ca^2+^-independent mechanisms^2,35,36,39^. The Ca^2+^-dependent effect of PKC could be mediated via Ca^2+^ entry, with the participation of w-agatoxin-TK-sensitive, Cav2.1-like, Ca^2+^ channels or possibly the non-selection cation channel^20,40^. Ca^2+^ could act via activation of the scramblase^36^. The present findings using the novel compound, Yoda1, are largely in agreement with these models. They are therefore consistent with Yoda1 acting as a PKC activator, as well as via PIEZO1 channels.

These previous reports using PMA and LPA^2,35^ failed to show a clear correlation between red cells with elevations in Ca^2+^ and PS exposure. They also suggested that PS exposure could not occur in the absence of extracellular Ca^2+^^2^, unlike the present findings. There is an important caveat here in that the high Ca^2+^ affinity of the fluorophores used (fluo3/4) is such that cells would show positive for Ca^2+^ at low submicrolar concentrations which may be insufficient to cause Ca^2+^-induced PS scrambling, which occurs at an EC_50_ of about 1 μM^6,18,37^. In addition, variable quenching of the fluorophore, known to be mediated by haemoglobin, may cause cells with similar Ca^2+^ levels to test negative.

Using Yoda1-induced PS exposure, the present results clearly indicate that this compound can elicit PS exposure in a dose-dependent manner in the complete absence of Ca^2+^ (Figs. 1, 2 and 3). They show that PKC inhibition prevents Yoda1-induced PS exposure in the absence of Ca^2+^ and a low [Ca^2+^]_i_, indicative of mediation via this enzyme. Using Ca^2+^ clamping with ionophore, they also show that Ca^2+^ and Yoda1 interact such that Yoda1 shifts the EC_50_ for Ca^2+^-induced PS exposure to lower values. Finally, they also show that high Ca^2+^ can overcome PKC inhibition (Fig. 7b), presumably through direct effects on the scramblase, but probably only at concentrations which would damage the cell in other ways. Notwithstanding, an important limitation of the present work is the use of inhibitors, rather than a molecular approach. Definitive proof of our conclusions must await genetic or molecular studies, perhaps using the CRISPR/Cas9 approach in immortalized red cells precursors.

Taken together the present findings provide further evidence for the dual role of PKC and Ca^2+^ in mediation of PS exposure in red cells from SCA patients.
